# How to Practice Dentistry Successfully

**Published:** 1893-01

**Authors:** J. L. Sweetnam

**Affiliations:** Manistee, Michigan


### How to Practice Dentistry Successfully.

BY J. L. SWEETNAM D.D.S., MANISTEE, MICHIGAN.

Read before the Michigan State Dental Society.

I have chosen the above title for a paper, for a two-fold reason, the first being the fact that it is the first time I have been called upon to read a paper by this Society, and if I am a successful practitioner, I ought to be able to present a few hints, that would not be entirely valueless.

Second, because many things could properly be said that might be of interest to this Association without putting forth any pet theory. To be successful, a dentist must command the confidence and respect of the people. In order to do this he must be the master of his calling, and, in addition to that, he must be honest, and cleanly in his habits.

Let us, then, consider for a few moments, first: What is meant by being " the master of his calling." Of course, we all appreciate the importance of a good education as a primary requisite, yet how many there are who take into their offices, as students, young men who are not qualified (by reason of their lack of education) to enter upon the technical study of dentistry. This is not only true of regular practitioners, but also of our dental schools, and, in my judgment, is the channel through which so many incompetent men have entered the rank and file of the dental profession. This evil is not entirely corrected, although it is growing less every year. I do not wish to be understood as meaning that a man should be required to hold an art's degree before being ready to enter upon the study of dentistry; if he can receive education to that standard, all the better, but a student should not be permitted to take up the study of dentistry, either in an office or dental college, until he, at least, has received a high-school education.

After a student has received a high-school education, he is sufficiently developed to comprehend written language. He will have a clear intellectual comprehension of what he reads; his mind will be drawn out to a point of self-reasoning, and he can eliminate from the advanced writers of science and literature that which will not stand the crucial test of investigation. After completing the curriculum of a dental college, he is in position to keep up with the advancement being made, and if he is honest he will do so.

Before passing the question of education, permit a few remarks on the salutary effect of dental society meetings. There are many young men who, through stress of circumstances, and, perchance, the misdirection of an unscrupulous and indifferent preceptor, enter the dental profession through the back door, many of whom have great ability and the desire to become proficient. Through the kind invitations of dental societies, they find their way to the conventions, and are made welcome by the extension of the right hand of fellowship. They are encouraged, and an interest shown in them makes them feel at home. They consequently become interested and go home with new and valuable ideas, and in many cases become more of an honor to the profession than their more fortunate brothers who have had better advantages. How important it is for us, then, to do what we can to contribute to the interest of the meetings, by our presence, and, if called upon to write a paper! Do it from a sense of duty, not because we think ourselves more competent than some one else, but because we are willing to do what we can. Let us not be parasites; we can all, at least, take part in the discussion of papers. We are apt to lose sight of the fact that conversational discussion is one of the very best means of acquiring knowledge. Plato grasped this truth, in fact his very theory of knowledge is a process of elimination from the accidental and fleeting to that which is logical. He called this process dialectics, and dialectics originally signified only conversational discussions.

Here, then, we have the means at hand to acquire knowledge in our particular calling, and it should be made use of. I grant that it is not an easy thing for a man to get up and take part in a meeting of any kind without experience in this sort of thing; but how can we get experience unless we try? The timid, therefore, ought to be encouraged, and I am glad to say they are encouraged by this Association.

Let us go back, then, and consider, in the next place, what is meant by "being honest. Granted that a dentist is thoroughly qualified and competent to do his patients good service, if he is dishonest he will slight many things that his judgment would tell him should be well done. For instance, he is about to insert a filling in the mesial surface of a bicuspid. After opening the cavity from the grinding surface, the meso-buccal and meso-lingual walls are found quite strong, with a good support of sound dentine. Now if that man is not honest he will fill the cavity without removing sufficient of these walls to give free margins to the filling, and every good operator knows the result. Then there are grooves and crevices that our conscience would dictate, should be cut out, but if dishonest they are permitted to remain untouched. Again, in the case of a gold crown or any band crown, we know it is not an easy thing to always get a nice adjustment at the neck of the tooth. On account of the conical shape of the root there is a tendency to leave a shoulder, or projection of the free end. An honest man will never permit such a piece of work to go out of his office.

I have said, if a man was honest he would keep up with the times; I mean, by that, that he should be acquainted with the latest methods and improvements, and should be posted on the current dental literature of the day. If he has done that there is no reason for his not knowing what to do in the management of enamel margins, after reading such able and very valuable series of papers as those of Drs. Black and Ottolengui, published last year in *The Dental Cosmos.* I confess that if I could have had the opportunity of reading such articles ten or twelve years ago, they would have saved many a good filling that was lost for the reason that I was afraid I should cut away a little sound tooth substance.

Then, we must be honest in our charges for services rendered. This is an important point; there are so many ways of reckoning the value of our services. In my judgment there is but one equitable way of making a charge, that is, by the length of time required to perform the operation. I would not have all members of the dental profession charge the same fee; there are all degrees of skill and should be a difference in compensation. Then let us charge a certain price per hour, and let that price be known to our patients. For instance, if I charge two dollars per hour for my services, it ought not make any difference to me if there is another dentist in the same city who is willing to work for one dollar per hour. I am not doing my patients an injustice, because some other man can not charge the same fee as I do and get patronage. Neither should it make any difference to me if there are dentists in larger cities who charge five or ten dollars per hour for their services; their expenses are larger, and it has taken time to build up a reputation that will enable them to command a high fee. I say, fix a definite price, per hour, for your services, and charge no more than that price, and your patients will not grumble; they know what to expect, and if they cannot afford your price, they have the privilege to look up a cheaper man. No one man ought to expect to hold the patronage of an entire community where there are other dentists.

We should be careful about our advice to patients; it is dishonest to advise an expensive piece of work when we know it will be only of temporary service.

Last, but not least, a few remarks about cleanliness. In the first place, we must be clean about our person ; a fine suit of clothes and clean linen will not fill the bill ; our person must be clean. If we take manual exercise as we should, we must bathe often enough to keep a sweaty smell from our body. It is important that the breath should be watched ; it is awful to go to work over a patient with a foul breath. If we use tobacco, limit the use until after office hours. The hands should be kept soft and clean, and our patient ought to have the satisfaction of seeing them washed the last thing before going to work. Now, it is not necessary to go through a lot of red tape, and do it in a sort of "Miss Nancy" way, rather do it as a matter of course, and in a business way. Then, the office should be kept in the best of order, and clean as the parlor of any patient we wish to do business for. The instruments should not only be clean, but look clean ; if they are nickel-plated, it is an easy matter to keep them looking nicely, and it should be done.

Enough of this ! We all know the importance of cleanliness in the dental office.

This is truly a period of transition, in dentistry as well as in many other matters. Then let us be alive to what is going on about us. I have tried to sketch, in a general way, those things of primary importance that should have our attention, rather than advocate any pet theories. A great deal more might be said, but there are a great many here to say it.

